# 566. The Impact of a Clinical Practice Guideline and Educational Interventions on the Management of Children Hospitalized with Uncomplicated Community Acquired Pneumonia

**DOI:** 10.1093/ofid/ofac492.619

**Published:** 2022-12-15

**Authors:** Sejal M Bhavsar, Tamanna Jabeen, Kristen Ramones, Katharine N Clouser, Jasmine Gadhavi, Pooja Shah, Donna J Lee, Rachel Lewis, Sondra M Nemetski

**Affiliations:** Hackensack University Medical Center, Hackensack, New Jersey; Hackensack University Medical Center, Hackensack, New Jersey; Hackensack University Medical Center, Hackensack, New Jersey; Hackensack University Medical Center, Hackensack, New Jersey; Hackensack University Medical Center, Hackensack, New Jersey; Rutgers University/Hackensack University Medical Center, Hackensack, New Jersey; Hackensack University Medical Center, Hackensack, New Jersey; Hackensack University Medical Center, Hackensack, New Jersey; Hackensack University Medical Center, Hackensack, New Jersey

## Abstract

**Background:**

The 2011 guidelines for management of pediatric uncomplicated community acquired pneumonia (CAP) recommend the use of ampicillin or penicillin first line. We sought to evaluate improvement in adherence to these guidelines through antimicrobial stewardship interventions at a single institution to minimize unnecessary broad antimicrobials.

**Methods:**

A retrospective chart review was conducted of admitted patients aged 2 months - 21 years old with uncomplicated CAP. The pre-intervention phase was September 1, 2019-February 29, 2020 and the post-intervention phase included September 1-February 28 of 2021 and 2022. Antimicrobial stewardship interventions included the incorporation of clinical practice guidelines (CPG) into a new institutional CAP pathway (approved in June 2020) and subsequent education to ordering providers in the spring of 2020. Patients with complicated pneumonia or with comorbidities including sickle cell disease, chronic lung disease, neurologic conditions, congenital heart disease or patients who were immunocompromised were excluded. The prescribing patterns of specific physicians were recorded and adherence to CPG recommendations were assessed. The primary endpoint was to measure the reduction of broad spectrum antibiotics (vancomycin, clindamycin, ceftriaxone, levofloxacin and cefdinir) to narrow spectrum antibiotics (ampicillin, amoxicillin, and amoxicillin-clavulanate).

**Results:**

A total of 114 patients were included in the study; 72 pre-intervention and 42 post-intervention. Mean age was 5.4 years pre- and 6.5 years post-intervention. A significant reduction in broad spectrum antibiotic use was noted in the ED (p=< 0.0001), during the first 24 hours of admission (*p*=0.0034) and for discharge antibiotics (*p*=0.0003) (Figures 1-3) between the pre- and post-intervention groups. Guideline adherence was 78.5%. No change in length of stay or treatment failure were observed.

Antibiotic Prescriptions in the Emergency Department

**Figure:**
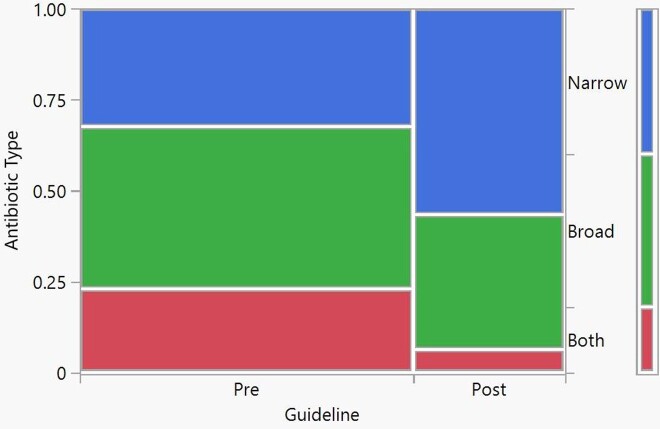

After the implementation of the CPG guideline there was a 17% reduction in the number of times a combination of antibiotics were used, a 7.2% reduction of broad spectrum antibiotics and a 24% increase in the utilization of narrow spectrum antibiotics, p-value= <0.0001.

Antibiotic Utilization 24 hours After Hospital Admission

**Figure:**
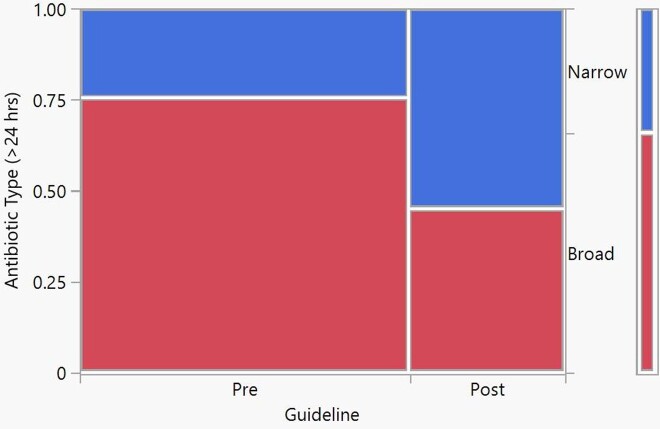

After the implementation of the CPG guideline there was a 20% reduction in the number of times a broad spectrum antibiotic was prescribed, p-value =0.0034.

Discharge Antibiotic Prescriptions

**Figure:**
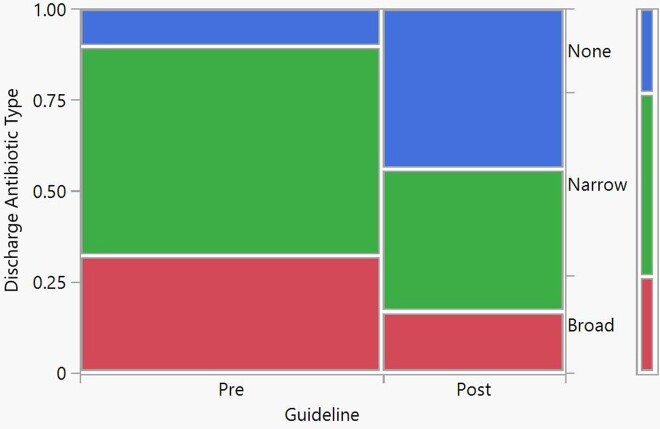

After the implementation of the CPG guideline there was a 18% reduction of broad spectrum antibiotics and a 34% increase in the utilization of narrow spectrum antibiotics, p=0.0003.

**Conclusion:**

The CPG and educational interventions had a positive impact on the antibiotic management of children hospitalized, with an overall 4.7% reduction of broad spectrum antibiotics and a 28% increase in the utilization of narrow spectrum antibiotics. Continued education may improve CPG adherence.

**Disclosures:**

**All Authors**: No reported disclosures.

